# Methotrexate-loaded multifunctional nanoparticles with near-infrared irradiation for the treatment of rheumatoid arthritis

**DOI:** 10.1186/s13075-020-02230-y

**Published:** 2020-06-18

**Authors:** You-Jung Ha, Sun-Mi Lee, Chin Hee Mun, Hyung Joon Kim, Yonghee Bae, Ji-Hee Lim, Kyu-Hyung Park, Soo-Kon Lee, Kyung-Hwa Yoo, Yong-Beom Park

**Affiliations:** 1 Division of Rheumatology, Department of Internal Medicine, Institute for Immunology and Immunologic Diseases, Yonsei University College of Medicine, 50, Yonsei-ro, Seodaemun-gu, Seoul, 03722 Republic of Korea; 2 Nanomedical Graduate Program, Yonsei University, Seoul, Republic of Korea; 3 Department of Physics, Yonsei University, 50, Yonsei-ro, Seodaemun-gu, Seoul, 03722 Republic of Korea

**Keywords:** Rheumatoid arthritis, Multifunctional nanoparticle, Photothermally controlled drug delivery, Methotrexate

## Abstract

**Backgrounds:**

Despite the advances of rheumatoid arthritis (RA) therapeutics, several patients do not receive adequate treatment due to the toxicity and/or insufficient response of drugs. The aim of this study is to design photothermally controlled drug release from multifunctional nanoparticles (MNPs) at a near-infrared (NIR) irradiated site to improve therapeutic efficacy for RA and reduce side effects.

**Methods:**

Au film was deposited onto methotrexate (MTX)-loaded poly(ethylene glycol)-poly(lactic-co-glycolic acid) (PLGA) nanoparticles, resulting in MTX-loaded MNPs. The synergistic effects of MTX-loaded MNPs with NIR irradiation were investigated using RA fibroblast-like synoviocytes (FLSs) and collagen-induced arthritis (CIA) mice.

**Results:**

Upon NIR irradiation, NIR resonance of the Au half-shell generated heat locally, accelerating MTX release from PLGA nanoparticles. In vivo NIR images of MTX-loaded MNPs indicated effective delivery of the MNPs to the inflamed joints. Moreover, in collagen-induced arthritis mice, MTX-loaded MNPs containing 1/1400 of MTX solution (repeated-dose administration) had therapeutic effects comparable to conventional treatment with MTX solution. In vitro experiments showed higher therapeutic efficacy of MTX-loaded MNPs with NIR irradiation than that of chemotherapy alone.

**Conclusions:**

A combination therapy of MTX-loaded MNP and NIR irradiation showed durable and good treatment efficacy for the suppression of arthritis in a single administration of small dose of MTX. Our results demonstrate that the treatment modality using drug-loaded MNP with NIR irradiation may be a promising therapeutic strategy for the treatment of RA and allow in vivo NIR optical imaging.

## Background

Rheumatoid arthritis (RA) is a chronic inflammatory disease characterized by synovial hyperplasia and bony erosion, leading to bone destruction and disability [1]. The mainstay of RA treatment is the administration of disease-modifying anti-rheumatic drugs (DMARDs) aimed at improving inflammation and retarding disease progression [2]. Methotrexate (MTX) is a structural analogue of folic acid that can competitively inhibit the binding of dihydrofolic acid to the enzyme dihydrofolate reductase, consequently inhibiting DNA formation and cell proliferation. MTX has been widely used as a DMARD and exhibits excellent efficacy and an acceptable toxicity profile [3, 4]. However, approximately 30% of RA patients cannot tolerate MTX because of its side effects, which include nausea, vomiting, stomatitis, hepatotoxicity, and bone marrow suppression [5]. The adverse effects of MTX are dose-dependent, so several delivery systems using nanoparticles (NPs), liposomes, microspheres, and different polymers have been suggested to improve the delivery of MTX [6].

Previously, we reported the use of multifunctional nanoparticles (MNPs) consisting of arginine-glycine-aspartic acid (RGD)-conjugated MTX-loaded poly(DL-lactic-co-glycolic acid; PLGA) Au half-shell NPs (RGD-MTX-PLGA-Au H-S NPs) [7]. RGD binds to a variety of integrins that are highly expressed in RA synovial tissue, so it serves as a targeting moiety for inflammatory sites, leading to enhanced drug delivery into inflamed synovium [8, 9]. However, the use of RGD may hinder clinical applications because of high costs and regulatory hurdles. Hence, we synthesized MTX-loaded MNPs without RGD and evaluated their passive targeting and therapeutic efficacy using a collagen-induced arthritis (CIA) mouse model. To confirm a synergistic effect of MTX-loaded MNPs combined with near-infrared (NIR) irradiation, we also investigated the therapeutic efficacy of MTX-loaded MNPs combined with NIR irradiation in RA fibroblast-like synoviocytes (FLSs).

## Methods

### Materials

PLGA (L:G molar ratio = 50:50; MW = 20,000) was purchased from Wako (Japan). MTX and Pluronic F-127 were purchased from Sigma-Aldrich Chemical Co. (St. Louis, MO, USA). Thiol-terminated methoxy-poly(ethylene glycol) (SH-PEG-OCH_3_, MW = 5000) was purchased from Creative PEG-Works (USA).

### Synthesis and characteristics of MTX-loaded MNPs

**Fig. 1:**
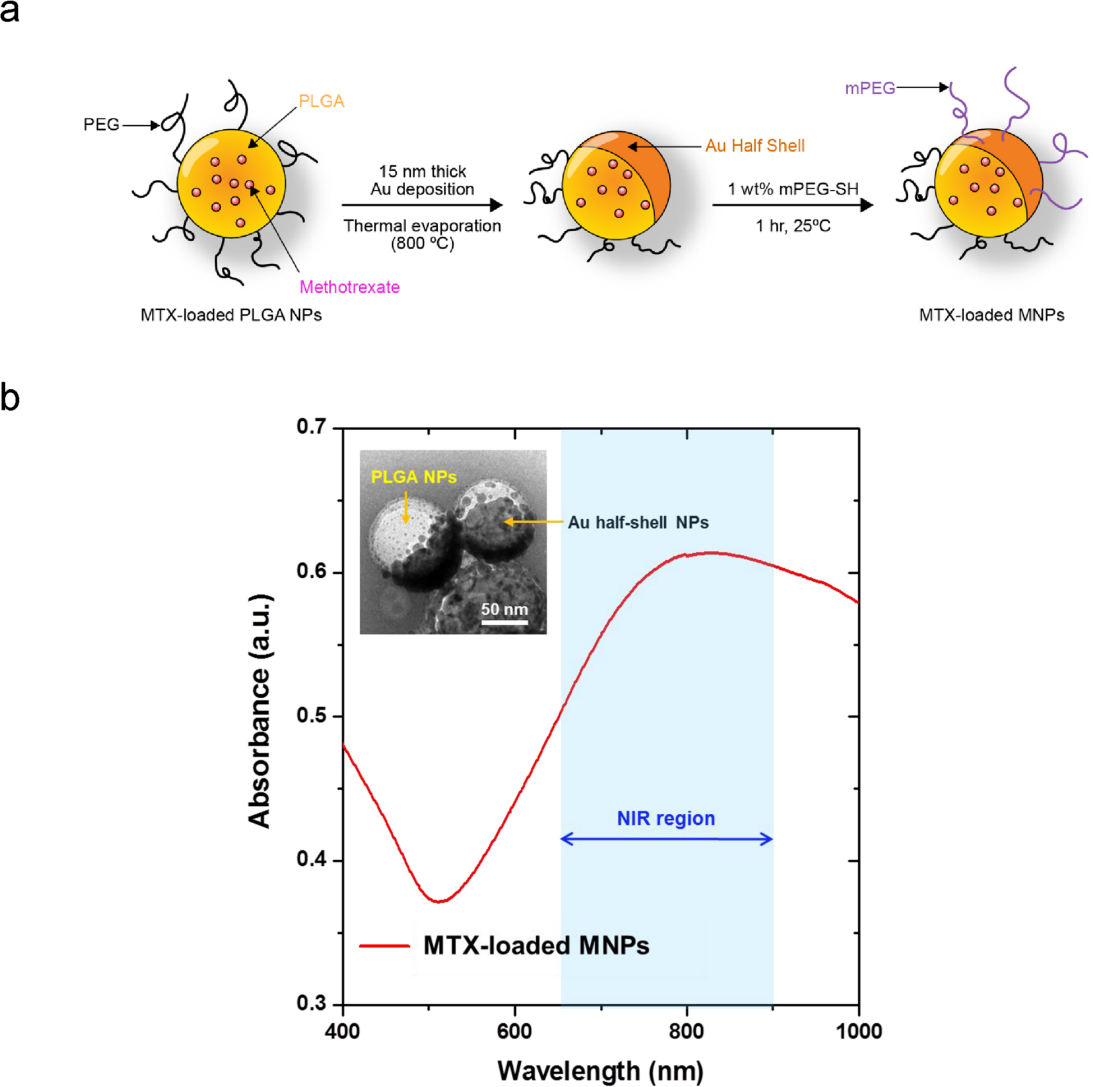
Fabrication and characteristics of MTX-loaded MNPs. **a** Schematic fabrication process of MTX-loaded MNPs. **b** TEM images of 100–115-nm MNPs with Au half-shell (left upper corner) and NIR absorption spectrum of the MTX-loaded MNPs

The MTX-loaded PLGA NPs were prepared as reported previously [10, 11]. Briefly, MTX (6 mg, Sigma-Aldrich) and PLGA (200 mg) were dissolved in dichloroethane (20 ml), which was stirred for 24 h at room temperature under nitrogen atmosphere. Then, the organic solution was slowly added drop-wise into distilled water (200 ml) containing Pluronic F-127 (2 mg) under magnetic stirring. The NPs were formed immediately, and the solvent was removed by overnight evaporation at room temperature. Then, the MTX-loaded PLGA NPs were collected by centrifugation and re-dispersed in distilled water (5 ml) by sonication. Au film (15 nm) was deposited onto an MTX-loaded PLGA NP monolayer prepared on a Si substrate using a thermal evaporator. To stabilize the NPs under in vivo conditions, Au-deposited MTX-loaded PLGA NPs were released from the substrate surface by sonication into 1 wt% thiol-terminated methoxy-PEG solutions and then collected by centrifugation, resulting in MTX-loaded MNPs with an Au half-shell structure (Fig. 1a). The Au half-shell structure of MTX-loaded MNPs was confirmed using a transmission electron microscope (TEM) (inset in Fig. 1b). The MTX loading efficiency, defined as the ratio of the actual mass of the MTX-loaded MNPs relative to the total mass of the MTX-unloaded MNPs, was estimated to be approximately 2.5%. Using dynamic light scattering, the MNPs were determined to be 100–115 nm in diameter (Additional file 1). The ultraviolet-visible/NIR absorption spectrum of the MTX-loaded MNPs exhibited a pronounced peak at approximately 810 nm due to the Au half-shells (Fig. 1b), suggesting that MTX-loaded MNPs can be used for photothermal treatment and in vivo NIR absorbance imaging.

### Immunofluorescence

Rhodamine-loaded MNPs were synthesized using a procedure similar to that for the MTX-loaded MNPs. A total of 200 μl of rhodamine solution was injected into the two hind foot pad of CIA mice. The same volume of rhodamine-loaded MNP solution (1 mg/ml dispersed in phosphate-buffered saline [PBS]) was injected into the four hind foot pad of other CIA mice. Subsequently, the limbs of two mice were exposed to NIR at 1.96 W/cm^2^, while the other two mice were unexposed. The amount of rhodamine in rhodamine-loaded MNPs was about 1/1200 of rhodamine solution. One mouse from each group was sacrificed 1 h after injection, and the other was sacrificed 24 h after injection. All samples were counterstained with 4′, 6-diamidino-2-phenylindole (DAPI). The paws were embedded in OCT compound and horizontally sectioned.

### Biodistribution

MTX-loaded MNPs (200 μl, 1 mg/ml dispersed in PBS) were administered intravenously (IV) into CIA mice (total *n* = 9). The mice were sacrificed 1, 3, or 28 days after injection (*n* = 3 at each point), and the major organs (liver, heart, spleen, kidney, and lung) and joints were removed from each mouse. Tissue samples were placed in a mixed acid matrix of *aqua regia* and heated overnight at 80–90 °C. After additional heating at 130–140 °C for 2 h, the organic compounds were completely removed and only ionized Au remained. This residue was dissolved in 1 ml of 0.5 M HCl and analyzed using an inductively coupled plasma mass spectrometer (ICP-MS, Agilent 7500C).

### Ex vivo NIR imaging

MTX-loaded MNPs (200 μl, 1 mg/ml dispersed in PBS) were injected intravenously into the CIA mice. The mice were sacrificed 1, 3, or 28 days after injection, and the major organs (liver, heart, spleen, kidney, and lung) were removed from each mouse. NIR absorbance images were obtained using an eXplore Optix System (Advanced Research Technologies Inc., Montreal, Canada) with the black color denoting the strongest absorption. Absorbance at 710~750 nm was detected with a fast photomultiplier tube (Hamamatsu, Japan) and a time-correlated single photon counting system (Becker and Hickl GmbH, Berlin, Germany). All data were calculated using the region-of-interest function of the analytical workstation software.

### CIA induction and assessment

All animal experiments were approved by and conducted in accordance with the regulations of the Institutional Animal Care and Use Committee of Yonsei University, Seoul, Korea. All mice were maintained in a specific pathogen-free facility. First, an intradermal injection of 200 μg bovine type II collagen (CII, Chondrex, Redmond, WA, USA) emulsified in 200 μg of complete Freund’s adjuvant (Chondrex, Redmond, WA, USA) was administered in the base of the tail of male DBA/1J mice (8 weeks old, Central Lab Animal, Inc., Seoul, Korea) to induce arthritis. Next, a booster intradermal injection of 100 μg bovine CII in incomplete Freund’s was given to mice at 14 days after the primary immunization. Mice were monitored twice a week for clinical assessment of arthritis. Disease severity was assessed in each limb according to a previously described scoring system (score 0–4) [12]. The scores for each limb were added, giving a maximum score of 16 per animal. Arthritis scoring was performed by two independent observers. Paw thickness was also measured twice a week with a Vernier caliper.

Upon arthritis development (arthritis score of 8–10), mice were randomly assigned to each treatment group (*n =* 5) at 4 weeks after the second booster injection, as described in Table 1. Briefly, saline (G1, positive control), low-dose MTX (G3), or PLGA-MNPs (G4–6) were once administered via intravenous tail injection. MTX-unloaded MNPs were given to G4, whereas MTX-loaded MNPs were given to G5 and G6. G4 and G6 were also exposed to 1.96 W/cm^2^ NIR light for 10 min the next day after MNP injection. A high-dose MTX solution group (G2, representing conventional MTX treatment) received intraperitoneal injection of 35 mg/kg MTX twice a week, weekly till sacrifice.

**Table 1:** Treatments applied to RA-FLS or CIA mice for comparative study of therapeutic efficacy

Group	Treated content	In vivo		Group	Treated content	In vitro	
		MTX dosage (mg/kg) and administration route^a^	NIR light (W/cm^2^)^b^			MTX concentration (μM)^c^	NIR light (W/cm^2^)^d^
Group 1	Saline	–	–	Group 1	Saline	–	–
Group 2	MTX solution (high dose)	35 × 8 times, IP	–	Group 2	MTX solution (low dose)	0.13	–
Group 3	MTX solution (low dose)	0.2 × 1 time, IV	–	Group 3	MTX solution (high dose)	30	–
Group 4	MTX-unloaded MNPs	–, IV	1.96	Group 4	MTX-unloaded MNPs	–	–
Group 5	MTX-loaded MNPs	0.2, IV	–	Group 5	MTX-unloaded MNPs + NIR	–	0.38
Group 6	MTX-loaded MNPs	0.2, IV	1.96	Group 6	MTX-loaded MNPs	0.13	–
				Group 7	MTX-loaded MNPs + NIR	0.13	0.38

^a^Treated volume of MNPs; 30 μl/ml, MNP concentration of 1 mg/ml. ^b^The arthritic limb was exposed to NIR light for 10 min using a laser diode (*λ* = 808 nm) 24 h post-injection. ^c^Treated volume of MNPs; 30 μl/ml, MNP concentration of 1 mg/ml. ^d^The RA-FLS was exposed to NIR light for 10 min using a laser diode (*λ* = 808 nm) 24 h post-incubation

### Histological examination

Mice were anesthetized and sacrificed on day 28 after treatment. The front and hind paws (including carpus, tarsus, and knee joints) were removed, and the skin from the ends of the digits was removed as well. Samples were fixed, decalcified, and embedded in paraffin. Tissue sections were prepared and stained with hematoxylin and eosin stain. The histological scores for changes in synovial inflammation, bone erosion, and proliferation were evaluated as previously described [13]. All histological analyses were evaluated blindly by three independent examiners.

### Measurement of immunoglobulin G antibodies to type II collagen and serum cytokines

Serum was collected from animals in each group of mice and stored at − 70 °C until assayed. The levels of serum anti-CII immunoglobulin G (IgG) were determined using a commercially available ELISA kit (#6018, Chondrex, Redmond, WA, USA) according to the manufacturer’s instructions. Serum levels of inflammatory murine cytokines interleukin (IL)-6, IL-12p70, and tumor necrosis factor (TNF)-α were determined using commercial mouse IL-6, IL-12p70, and TNF-α enzyme-linked immunosorbent assay (ELISA) kits (BD Biosciences, USA). Each sample was assayed in duplicate.

### FLS preparation and culturing

Synovial tissue was obtained from three RA patients who underwent knee joint replacement surgery at Severance Hospital, Yonsei University College of Medicine, Seoul, Korea. The study was approved by the ethics committee of our institution (IRB No. 4-2019-0683), and written informed consent was obtained from all participants. All patients satisfied the 1987 revised American College of Rheumatology classification criteria for RA [14]. FLS cells were isolated from 3 different donors and cultured as previously described [15]. Cells obtained from passages 4 to 8 were seeded onto 12-well plates at a density of 2 × 10^4^ cells/well in Dulbecco’s modified Eagle’s medium supplemented with 10% fetal bovine serum (Gibco BRL, Grand Island, NY, USA), 2 m*M*l-glutamine, 100 units/ml of penicillin, and 100 g/ml of streptomycin. The seeded cells were incubated at 37 °C.

### Apoptotic cell assay

A fluorescein isothiocyanate (FITC) Annexin V Apoptosis Detection kit (BD Biosciences, San Diego, CA, USA) was used to quantify the apoptotic cells of cultured FLS, according to the manufacturer’s protocol. Cells were treated with MTX solution (0.13 or 30 μM), MTX-unloaded MNPs with or without NIR irradiation, and MTX-loaded MNPs (0.13 μM of MTX) with or without NIR irradiation and incubated at 37 °C for 48 h. NIR irradiation was conducted 24 h after treatment at 0.38 W/cm^2^ for 10 min. Treated cells were harvested by centrifugation and washed in cold PBS. Annexin V-FITC and propidium iodide (PI) were added to 400 μl of cells. Following a 15-min incubation in the dark at room temperature, the stained cells were immediately analyzed by flow cytometry (FACS Calibur, BD). A minimum of 10,000 events were collected and analyzed using FlowJo software (TreeStar, Inc., Ashland, OR, USA). Annexin V-positive and PI-negative cells were defined as early apoptotic cells, and PI-positive cells were defined as late apoptotic or necrotic cells.

### Statistical analyses

All statistical analyses were conducted using IBM SPSS Statistics, version 20 (SPSS Inc., Chicago, IL, USA). Data are shown as means ± standard deviation. Statistical analyses of group differences were performed using the Mann-Whitney *U* test or ANOVA followed by Tukey’s method. For all analyses, *P* values < 0.05 were considered statistically significant.

## Results

### Photothermally controlled drug release

**Fig. 2:**
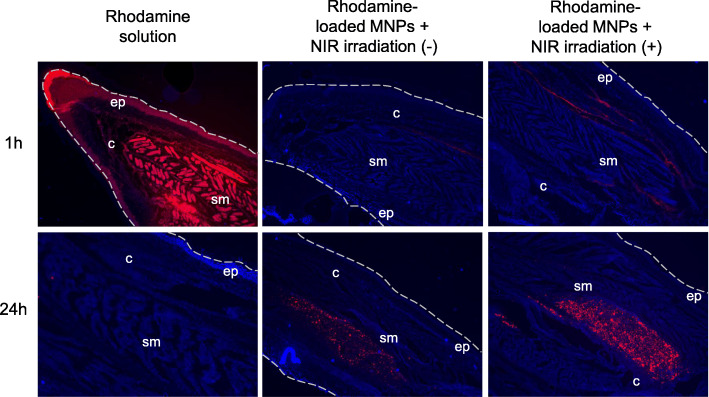
The local drug release effects of NIR irradiation on MNPs. The sections of the inflamed paw were examined at 1 h and 24 h after local administration of rhodamine solution and rhodamine-loaded MNPs without/with NIR irradiation. Sections were counterstained by DAPI (blue). Dotted lines indicate the boundaries of paws. c, connective tissue; ep, epidermis; sm, skeletal muscle. Original magnification × 100

To check local photothermal effect on drug release from MNPs, we injected 200 μl of rhodamine solution into a CIA mouse and acquired the optical images from the tissues that were sectioned from the inflamed paws at 1 h and 24 h after injection (Fig. 2 left panel). Rhodamine was prominently detected at 1 h, but not at 24 h, suggesting that rhodamine was rapidly cleared from the body. Next, we administered 200 μl of rhodamine-loaded MNPs (1 mg/ml dispersed in PBS) into the arthritic hind food pad of CIA mice. Figure 2 shows the optical images obtained from the inflamed paws unexposed (mid panel) and exposed to NIR light (right panel) for 10 min at 1.96 W/cm^2^ after treatments of rhodamine-loaded MNPs, respectively. Both mice showed rhodamine at 24 h, indicating that rhodamine-loaded MNPs were accumulated in the inflamed paw until 24 h. However, compared to the inflamed paw unexposed to NIR, the inflamed paw exposed to NIR yielded a higher intensity of rhodamine at 1 and 24 h due to an enhanced release rate of rhodamine caused by NIR irradiation.

### In vivo delivery and distribution of MNPs

**Fig. 3:**
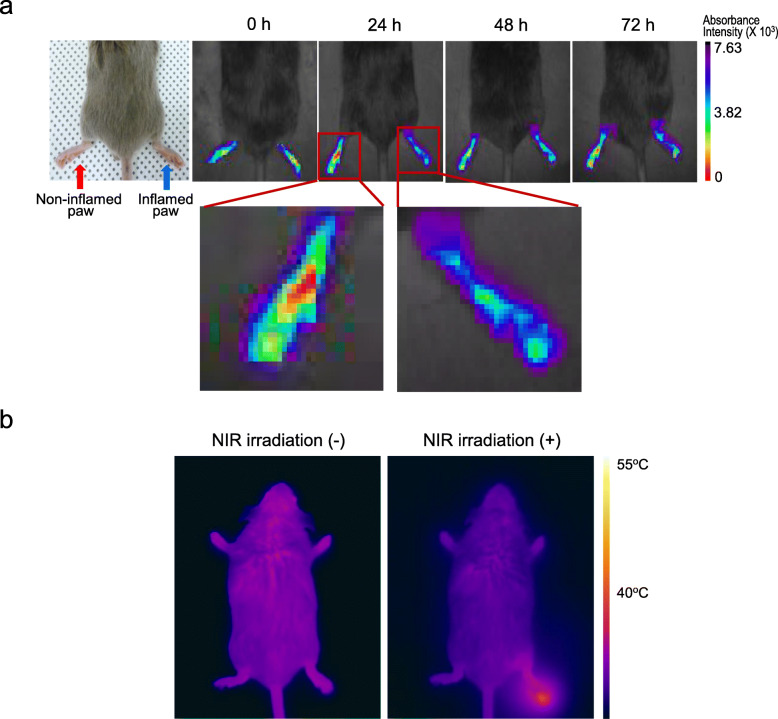
In vivo delivery and distribution of MTX-loaded MNPs. **a** In vivo NIR absorbance images of inflamed and non-inflamed paws of CIA mouse after intravenous injection of MTX-loaded MNPs (the picture below is a × 4 magnification of red box). **b** Thermal images of CIA mice treated with MTX-loaded MNPs, prior to and post NIR irradiation

To determine whether MTX-loaded MNPs are delivered to and accumulated in an inflamed region, 200 μl of MTX-loaded MNP solution (1 mg/ml dispersed in PBS) was administrated IV into a CIA mouse with an inflamed right paw and non-inflamed left paw. Time-lapse in vivo NIR absorbance images measured at 24 h after IV injection revealed that absorbance intensity ≥ 4 × 10^3^ a.u. (blue in Fig. 3a) increased over time in the inflamed paw whereas a change in the absorbance intensity was not apparent in the non-inflamed paw (Fig. 3a). These results indicated that the MTX-loaded MNPs were preferentially delivered to and accumulated in the inflamed paw.

In addition, the distribution of injected MTX-loaded MNPs (200 μl, 1 mg/ml dispersed in PBS) in major organs and inflamed joint was measured by ICP-MS at 1, 3, and 28 days following the IV injection (Additional file 2a). The MTX-loaded MNPs accumulated efficiently in the inflamed joint and demonstrated extended retention until 3 days; 0.16 μg of Au was found in the inflamed joints of CIA mice treated with MTX-loaded MNPs 24 h post administration, corresponding to 0.7% of the injected MTX-loaded MNPs. MTX-loaded MNPs were also taken up by the liver, lung, and spleen, with lesser accumulation in the kidney and heart. However, the amount of MTX-loaded MNPs in these organs was significantly reduced at 28 days. We also measured ex vivo NIR absorbance images of the injected MNPs and found a biodistribution similar to the ICP-MS results (Additional file 2b).

The left panel of Fig. 3b shows a thermal image of a CIA mouse treated with 24 h post-injection MTX-loaded MNPs (200 μl) and unexposed to NIR light. The temperature was approximately 36 °C throughout the entire body, corresponding to the body temperature of the mouse. However, when the inflamed paw was irradiated by NIR (1.96 W/cm^2^) for 10 min, the temperature increased to 43 °C only in the irradiated paw, and no significant temperature increase was observed in the region that was not exposed to NIR (right panel in Fig. 3b). These findings indicate that heat can be directed to only the inflamed region using laser guidance.

### Comparisons of therapeutic efficacy in CIA mice

**Fig. 4:**
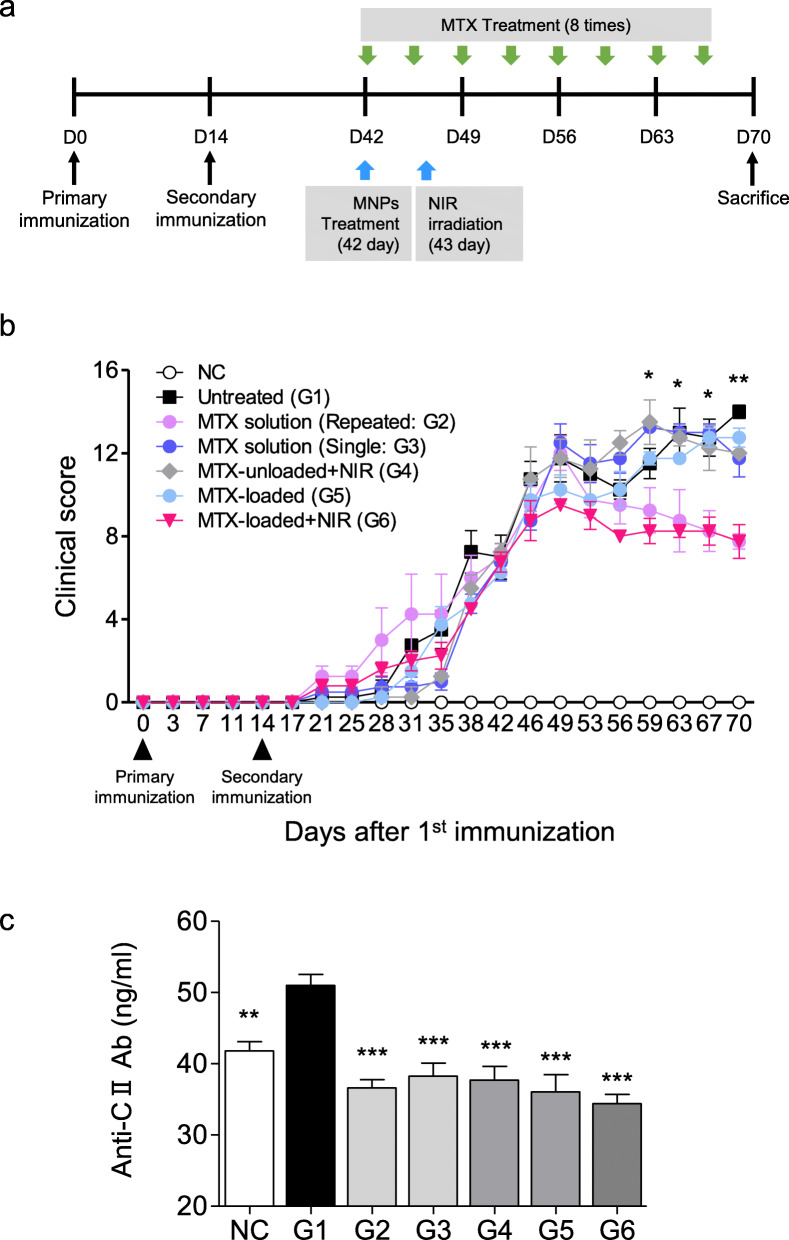
Comparative studies of therapeutic efficacy in CIA mice. **a** The schedule for CIA induction and treatment. **b** Severity of arthritis in CIA mice. NC, negative control; G1, PBS-treated; G2, 35 mg/kg MTX solution twice per week intraperitoneal; G3, 0.2 mg/kg MTX solution once IV; G4, MTX-unloaded MNPs IV + NIR irradiation; G5, MTX-loaded MNPs IV; G6, MTX-loaded MNPs IV + NIR irradiation. Mean clinical scores were significantly decreased in G2 and G6 mice compared with untreated mice. **c** Changes in anti-collagen II IgG titers in mice of different treatment groups. Data are expressed as mean ± SEM of 5 mice for duplicated serum samples. **P < 0.01, ***P < 0.001 vs. the untreated group (G1)

To investigate the therapeutic efficacy of MTX-loaded MNPs, thirty CIA mice were evenly divided into six groups (*n* = 5 per group), as summarized in Table 1. The timetable of CIA induction and applied treatment is described in Fig. 4a. Figure 4b shows the clinical index of these groups as a function of time. For a positive control, 35 mg/kg MTX solution was intraperitoneally administrated twice per week for 4 weeks, leading to a total MTX solution of 280 mg/kg (G2). The clinical index decreased from day 49, 1 week post treatment. G6 was treated with MTX-loaded MNPs (0.2 mg/kg of MTX) and exposed to NIR light (1.96 W/cm^2^) for 10 min at 1 day after MNP treatment. Despite a much lower MTX dosage for G6 (0.2 mg/kg) than G2 (280 mg/kg), similar treatment efficacy was obtained. However, when treated with 0.2 mg/kg MTX solution (G3) or MTX-unloaded MNPs with NIR irradiation (G4), arthritis was not ameliorated in mice although treatment with MTX-loaded MNPs without NIR irradiation (G5) led to a partial treatment only during the early period. These results demonstrate that chemo-photothermal treatment using MTX-loaded MNPs is a good way to maximize therapeutic efficacy and minimize dosage-related MTX side effects in the treatment of RA. It is well established that anti-CII antibody is involved in the pathogenesis of CIA [16]. Serological levels of CII-specific IgG were measured 4 weeks after treatment to determine whether the treatment was associated with a change in the humoral immune response to CII. As shown in Fig. 4c, the anti-CII IgG levels in the sera of G2-G6 mice were significantly reduced than those in G1 mice. Among them, the levels of serum anti-CII IgG decreased the most in G2 and G6.

**Fig. 5:**
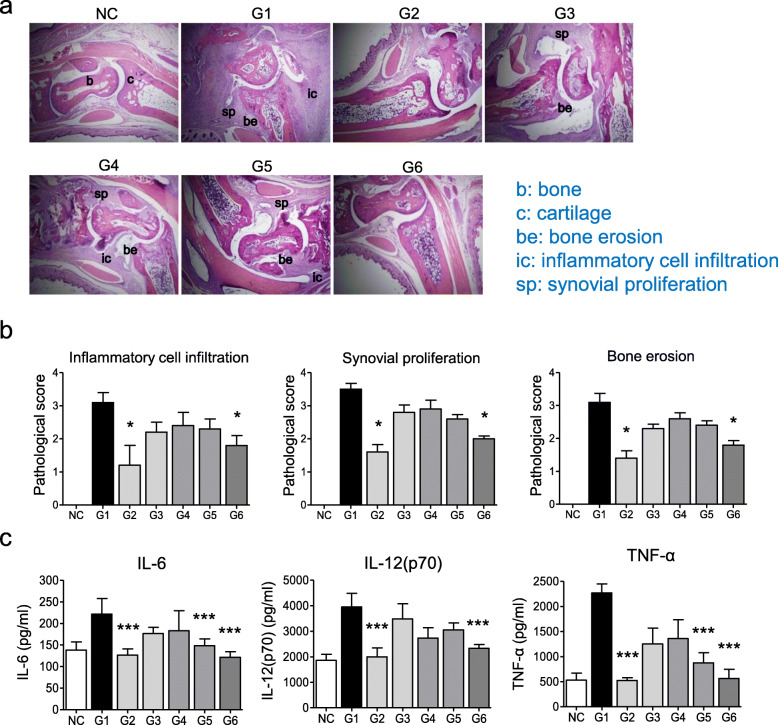
MTX-loaded MNPs with NIR irradiation ameliorate histopathologic changes and suppress pro-inflammatory cytokines in CIA mice. **a** Histological findings of ankle joints, original magnifications × 100. b, bone; c, cartilage; b, bone erosion; ic, inflammatory cell infiltration; sp, synovial proliferation. **b** Semiquantitative analyses of histological changes, *P < 0.01 vs. the untreated group (G1). **c** Cytokine assays by ELISA. *P < 0.05, **P < 0.01, ***P < 0.001 vs. the untreated group (G1). Values are expressed as mean ± SEM of five mice

Histopathological evaluation of the joint sections of untreated mice showed severe inflammatory cell infiltration, synovial hyperplasia, and bony erosion. These histopathological changes were significantly reduced in G2 and G6 mice. In contrast, no significant differences were observed in G3, G4, or G5 mice (Fig. 5a, b). To investigate whether MNPs could decrease serum levels of pro-inflammatory cytokines, we measured the levels of IL-6, IL-12p70, and TNF-α in the serum of CIA mice. Compared with untreated mice, the serum cytokine levels were significantly decreased in mice treated with 35 mg/kg MTX solution twice per week for 4 weeks (G2) and MTX-loaded MNPs with NIR irradiation (G6) (Fig. 5c). These results suggest that the therapeutic effect of MNPs with NIR irradiation is comparable to that of conventional MTX treatment.

### In vitro studies in RA-FLS with MTX-loaded MNPs

**Fig. 6:**
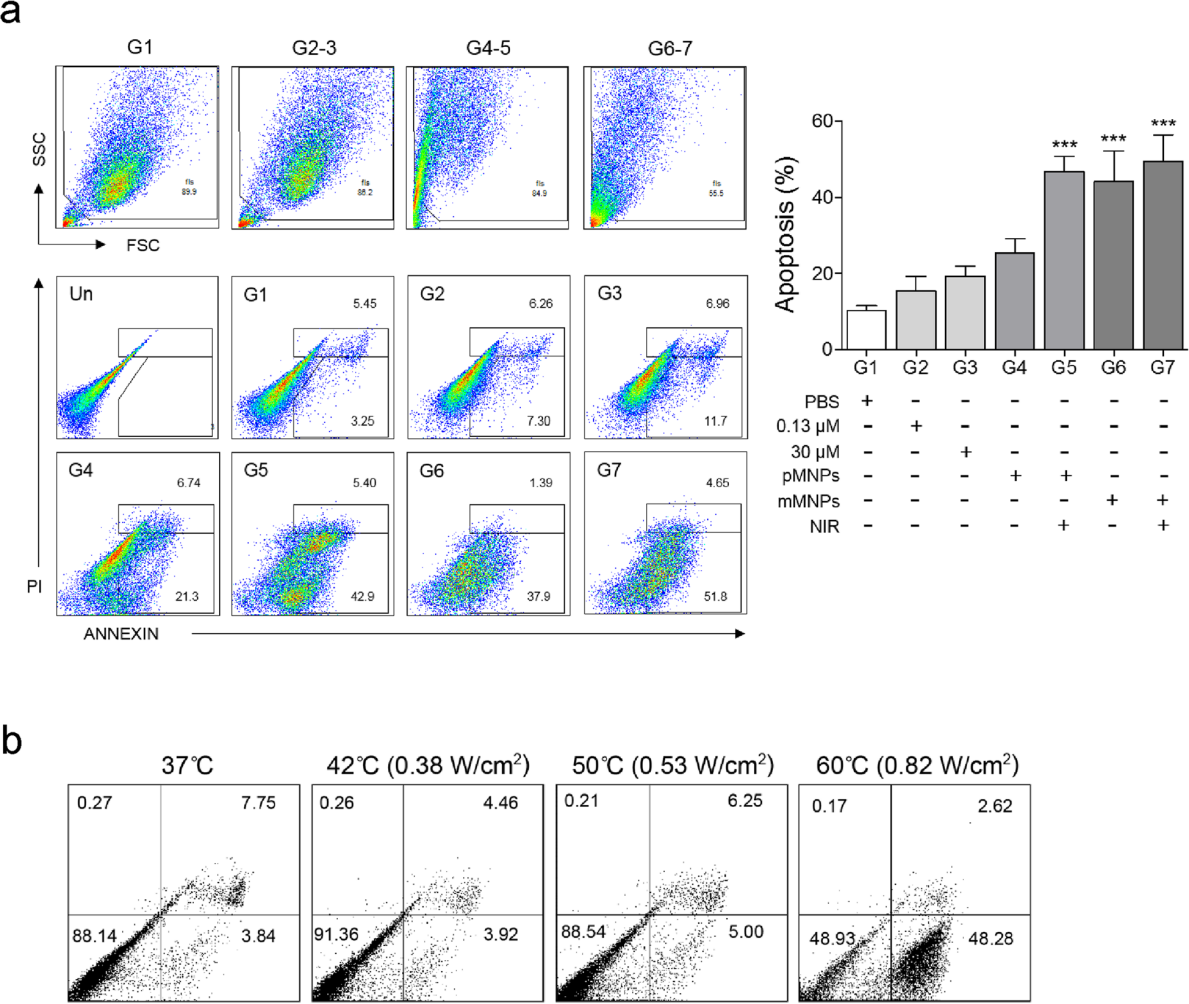
The in vitro effect of MTX-loaded MNPs and NIR irradiation. **a** Gating strategy (left upper) and representative dot plot images (left lower) and a graph (right) on induction of apoptotic death by each treatment in RA-FLS. RA-FLS cells were incubated with various treatments for 72 h and then analyzed by flow cytometry. PBS, PBS-treated FLS; 0.13 μM, MTX solution of 0.13 μM; 30 μM, MTX solution of 30 μM; pMNPs, MTX-unloaded MNPs; mMNPs, MTX-loaded MNPs; NIR, NIR irradiation. **b** Cell viability versus temperature during NIR irradiation for RA-FLS cells treated with MNPs. *P < 0.05, **P < 0.01, ***P < 0.001 vs. the control group. Values are expressed as mean ± SEM of each experiment. The experiments were conducted in duplicate

To study the anti-arthritic effect of MTX-loaded MNPs and NIR irradiation in vitro, we divided RA-FLS cells into seven groups and treated them differently, as summarized in Table 1. G1 was a FLS control group. G2 and G3 were treated with MTX solution of 0.13 μM and 30 μM, respectively. G4–G7 were prepared by culturing the cells with MTX-unloaded MNPs (G4 and G5) or MTX-loaded MNPs (MTX 0.13 μM; G6 and G7) for 1 day, followed by washing with PBS. After that, RA-FLS cells treated with MNPs were exposed to NIR for 10 min at 0.38 W/cm^2^ in G5 and G7, and apoptotic cell death was evaluated using a FACS at 48 h after NIR irradiation (Fig. 6a). As expected, G2 yielded much less apoptotic cell death than G3 because of the lower MTX concentration. The apoptotic cell death in G4 tended to be increased than that in G1, but the differences were insignificant, implying that MTX-unloaded MNPs alone did not significantly affect apoptotic cell death. Adding NIR irradiation to MTX-unloaded MNPs (G5) and treatment of MTX-loaded MNPs without NIR irradiation (G6) significantly enhance apoptotic cell death. The combined treatment of MTX-loaded MNPs and NIR irradiation (G7) lead the greatest apoptotic cell death, although the dosage of MTX in G7 was only 1/230 of that in G3. These results demonstrated a synergistic effect of MTX-loaded MNPs combined with NIR irradiation.

We also measured the cell viability of RA-FLS cells treated with MTX-unloaded MNPs and exposed to NIR light for 10 min at different powers to investigate the dependence of cell viability on temperature (Fig. 6b). At 0.82 W/cm^2^, the temperature increased to 60 °C, and the cell viability was reduced to about 49% due to cell death. However, for the NIR power less than 0.53 W/cm^2^, the cell viability remained higher than 88%, indicating that cell viability was not much affected by NIR irradiation at low powers.

## Discussion

We investigated the clinical potential of MTX-loaded MNPs with NIR irradiation for the treatment of RA. MTX-loaded MNPs showed more delivery to inflamed joints and induced sustained release, especially when irradiated with NIR. In vitro and in vivo studies using RA-FLSs and CIA mice confirmed photothermally controlled drug release and anti-arthritic effects of the combination therapy of MTX-loaded MNPs and NIR irradiation.

Although MTX is one of the most effective drugs for the management of RA, some limitations restrict the use of MTX. The pharmacokinetics of oral low-dose MTX appears to be variable and largely unpredictable even in patients with normal renal and hepatic function [17]. MTX has also several side effects such as gastrointestinal toxicities (stomatitis, nausea, and abdominal distress), alopecia, bone marrow suppression, and hepatotoxicity [18]. Approximately one third of RA patients discontinue MTX due to toxicity rather than lack of efficacy. Hence, there have been several attempts to develop novel MTX delivery systems in order to overcome these shortcomings. In this regard, nano-sized carriers are promising methods which can selectively deliver therapeutic agents to the inflammatory sites in a controlled or sustained manner [19, 20]. Various nanomaterials including human serum albumin conjugates, chitosan conjugates, liposomal conjugates, or in situ forming hydrogels of polymeric micelles have been suggested [6, 21]. Our polymeric MNPs also showed the selective delivery to the inflamed site and sustained release of contained drug and exhibited comparable efficacy in a small dosage when irradiated with NIR. These aspects of our MNPs could reduce the concerns about systemic toxicity of MTX.

Anti-inflammatory action of low-dose MTX for the treatment of RA has been related to the induction of apoptosis in part [22]. Our in vitro studies demonstrated that apoptotic death in RA-FLSs was induced by MTX-loaded MNPs and enhanced by NIR irradiation. Because the dose of MTX within MNPs was 0.13 μM MTX, MTX-loaded MNPs produced less apoptotic effect, compared with MTX 30 μM. However, when irradiated with NIR, the apoptotic effect by MTX-loaded MNPs was significantly increased. It is well-known that gold (Au) half-shells strongly absorb NIR light and act as heat carriers [23]. The cytotoxicity of methotrexate is enhanced by increasing the temperature [24], and another in vitro study has shown that MTX release from MTX-loaded gold nanoparticles was temperature-dependent [25]. These results imply that increased temperature of RA-FLSs treated with MNPs upon NIR irradiation may enhance the therapeutic effects of MTX.

In vivo studies using CIA mice revealed that MTX-loaded MNPs were delivered to the inflamed paw so that only the inflamed paw could be locally heated using the NIR laser diode. In addition, MTX-loaded MNPs were accumulated in the inflamed paw over an extended time and release of MTX was sustained for at least 3 days. The characteristics of inflamed RA synovium include abundant angiogenesis, abnormal and leaky vasculature, and an influx of inflammatory leukocytes [26]. Because MTX-loaded MNPs are administrated by the intravenous route, they can be more delivered to the hypervascular area (passive targeting). Also, the size of our NPs is attributable to targeted delivery, because 100–115-nm-sized nanoparticles are not much eliminated by the spleen and kidney [20]. Another explanation for selective passive targeting and sustained release of these MNPs without further integration of a specific targeting moiety might be due to locally enhanced permeability effects in the inflamed synovium [27, 28]. In the inflamed joints, rapid angiogenesis and large gaps between endothelial cells of blood vessels lead to selective extravasation of macromolecules. In the normal tissues, macromolecules are cleared rapidly via the lymphatic system, but the clearance of macromolecules in the inflammatory interstitium is relatively impaired that they can remain for a long time. The enhanced permeability effects of PLGA nanoparticle were previously reported in several studies [29].

To confirm the local photothermal effect on MNP of NIR irradiation, body temperatures were measured at different sites and conditions. Body temperatures were increased in the MNP-administrated and NIR-irradiated conditions, and that effect was remarkable in the inflamed area. Using rhodamine-loaded MNPs, we showed prolonged retention for incorporated chemical of MNPs in the NIR-irradiated areas. Therefore, co-application of MNP and NIR irradiation showed photothermal effect and more release of incorporated MTX, leading to better therapeutic effect in the NIR-irradiated area [30]. Thermotherapy is commonly employed means of non-pharmacological pain management in RA, although its scientific evidence for efficacy has not been strongly proven [31]. In addition, mild heating itself has been shown to decrease the joint stiffness in chronic arthritis [32]. Markovic et al. also demonstrated that short-term hyperthermia suppressed the activation of pro-inflammatory cytokine in RA-FLSs via blocking the activation of NF-κB [33]. Altogether, the application of NIR irradiation in patients with RA might be expected not only to enhance the therapeutic efficacy of administered MNPs but also to relieve the symptoms associated with inflammatory arthritis.

Our in vivo studies in the CIA mice model clearly showed that administration of MNPs with NIR irradiation (1) ameliorated the clinical signs of arthritis, (2) suppressed serum levels of pro-inflammatory cytokines and anti-CII IgG, and (3) reduced inflammation and prevent bone erosion in the joints despite the low MTX dosage. The therapeutic efficacy of the MNPs was similar to that observed in conventional MTX-treated CIA mice. Treatment with MTX at the same dosage as loading MTX in the MNPs (G3) did not ameliorate arthritis. Administration of MNPs without NIR irradiation was partially effective only during the early period, possibly because the absence of NIR irradiation hampered the sustained release of MTX.

Recently, several studies to investigate nanoparticles containing MTX for the RA treatment have been published. Reported materials include MTX-lipidic nanoemulsion, MTX-polysialic acid-trimethyl chitosan (PSA-TMC) NPs, MTX-superparamagnetic iron oxide PLGA NPs, N-PSA-TMC NPs coated with decoy oligodeoxynucleotides specific to transcription factor NF-κB, and MTX-loaded biodegradable NPs targeting CD34^+^ cells [34–38]. Among them, MTX-lipidic nanoemulsion was developed for intra-articular use, and other NPs were relatively complex to make. Unlike these materials, our MNPs are easy to make and have Au half-shells for local heating which enable the photothermal treatment.

## Conclusions

In the present study, we demonstrate the synergistic therapeutic effects of MTX-loaded MNPs and photothermal therapy for the treatment of RA. Combined with NIR irradiation, these MTX-loaded MNPs exhibited similar efficacy with a much lower dosage of MTX (approximately 1/1400) without a targeting ligand. Additionally, repetitive dosing with MTX-containing MNPs may enhance the therapeutic effect on arthritis; further studies will be required to examine these possibilities. Because of their relatively easy synthesis, low toxicity, and synergistic effect with NIR irradiation, these MNPs could have great potential for the treatment of RA. Furthermore, these MNP systems can be applied with other DMARDs.

## Supplementary information

**Additional file 1.** Characterization of prepared NPs.

**Additional file 2 **Delivery and accumulation of intravenously injected MTX-loaded MNPs. **a** Biodistribution of injected MTX-loaded MNPs measured by ICP-MS in major organs and joints (*n* = 3 at each time point). **b** Ex-vivo NIR absorbance images of injected MTX-loaded MNPs in CIA mice. *P < 0.05 vs. the 1 day group. Values are expressed as mean ± SEM of three mice.

## Data Availability

The datasets used and/or analyzed during the current study are available from the corresponding author on reasonable request.

## Abbreviations

RA: Rheumatoid arthritis
DMARDs: Disease-modifying anti-rheumatic drugs
MTX: Methotrexate
NP: Nanoparticle
MNP: Multifunctional nanoparticle
RGD: Arginine-glycine-aspartic acid
PLGA: Poly(DL-lactic-co-glycolic acid)
CIA: Collagen-induced arthritis
NIR: Near-infrared
FLS: Fibroblast-like synoviocyte
TEM: Transmission electron microscope
PBS: Phosphate-buffered saline
DAPI: 4′, 6′-Diamidino-2-phenylindole
IV: Intravenously
ICP-MS: Inductively coupled plasma mass spectrometer
CII: Type II collagen
IgG: Immunoglobulin G
IL: Interleukin
TNF: Tumor necrosis factor
ELISA: Enzyme-linked immunosorbent assay
FITC: Fluorescein isothiocyanate
PI: Propidium iodide
