# Supplementary material for: Methotrexate-loaded multifunctional nanoparticles with near-infrared irradiation for the treatment of rheumatoid arthritis
Source: Arthritis Res Ther. 2020 Jun 18;22:146. doi: 10.1186/s13075-020-02230-y (PMC7302395; doi:10.1186/s13075-020-02230-y)
Supplement: Supplementary file 1 — Additional file 1. Characterization of prepared NPs. [file 13075_2020_2230_MOESM1_ESM.docx]

**Additional file 1** Characterization of Prepared NPs

| Nanoparticles | Size [nm] ^[a]^ | PDI^[b]^ | Zeta-potential [mV]^[c]^ | MTX loading content [wt%]^[d]^ | Au thickness [nm] |
| --- | --- | --- | --- | --- | --- |
| MTX-PLGA NPs | 105 | 0.015 | -39.1± 1.34 | 3.52 ± 0.13 | - |
| MTX-loaded MNPs | 110 | 0.021 | -23.5± 1.21 | 3.02 ± 0.12 | 15 |

^[a,b,c]^The size, polydispersiy index (PDI) and zeta-potential of the NPs were measured by dynamic light scattering (Zetasizer Nano ZS, Malvem Instruments Ltd) at a concentration of approximately 1 mg of NPs/1 ml of distilled water. ^[d]^To determine the amount of encapsulated MTX, the dried NPs were redissolved in DMSO to extract MTX from the NPs. MTX in the supernatant was quantified by a UV spectrometer (Optizen 2120 UV, MECASYS Co.).
