# Supplementary material for: Methotrexate-loaded multifunctional nanoparticles with near-infrared irradiation for the treatment of rheumatoid arthritis
Source: Arthritis Res Ther. 2020 Jun 18;22:146. doi: 10.1186/s13075-020-02230-y (PMC7302395; doi:10.1186/s13075-020-02230-y)

**Additional file 2** Delivery and accumulation of intravenously injected MTX-loaded MNPs. **a** Biodistribution of injected MTX-loaded MNPs measured by ICP-MS in major organs and joints (*n* = 3 at each time point). **b** *Ex-vivo* NIR absorbance images of injected MTX-loaded MNPs in CIA mice. **P* < 0.05 *vs*. the 1 day group. Values are expressed as mean ± SEM of three mice.


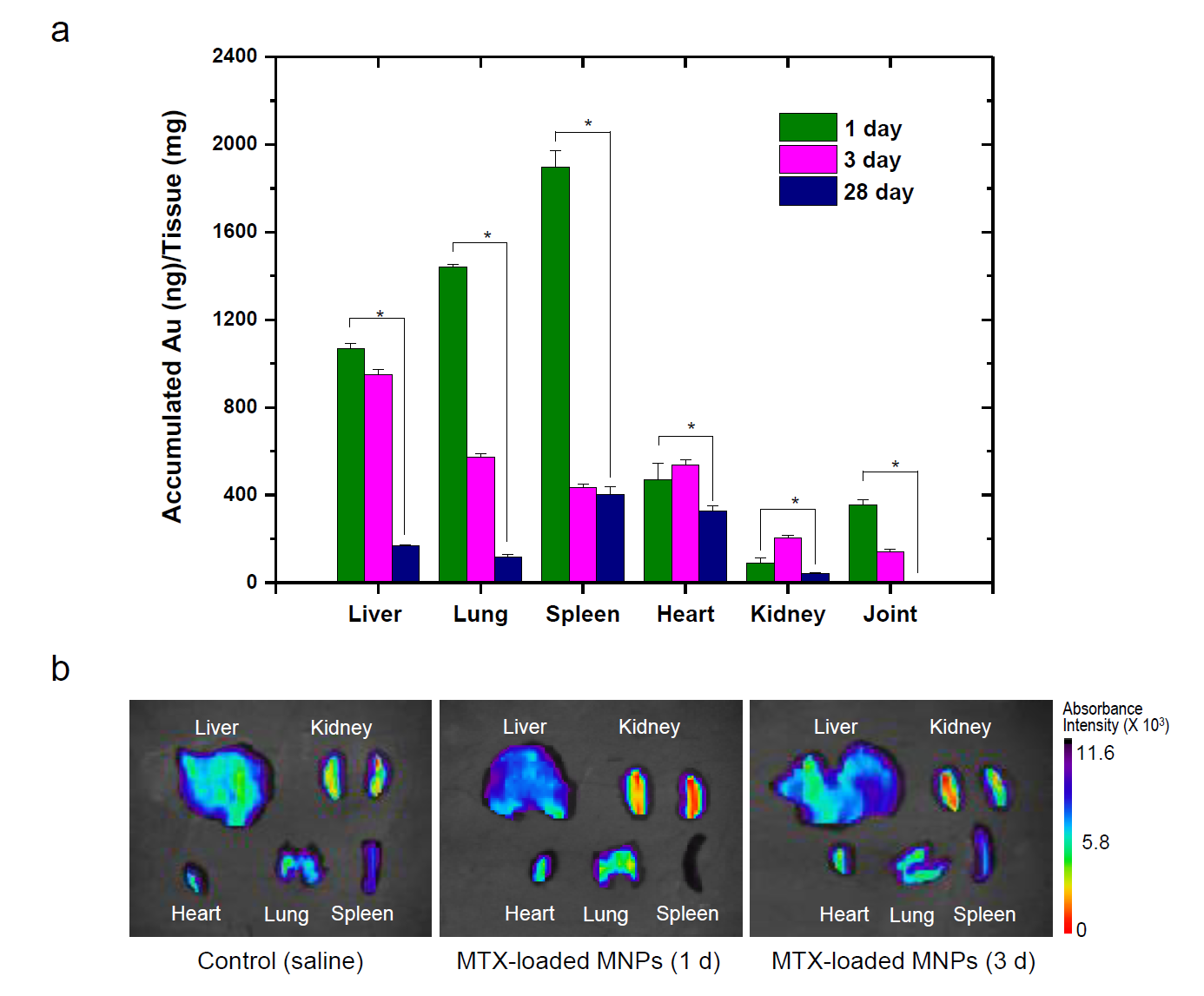

Supplement: Supplementary file 2 — Additional file 2 Delivery and accumulation of intravenously injected MTX-loaded MNPs. a Biodistribution of injected MTX-loaded MNPs measured by ICP-MS in major organs and joints (n = 3 at each time point). b Ex-vivo NIR absorbance images of injected MTX-loaded MNPs in CIA mice. *P < 0.05 vs. the 1 day group. Values are expressed as mean ± SEM of three mice. [file 13075_2020_2230_MOESM2_ESM.docx]
